# Membranoproliferative glomerulonephritis with deposition of monoclonal IgG evolved from polyclonal IgG: a case report with two consecutive renal biopsies

**DOI:** 10.1186/s12882-019-1453-4

**Published:** 2019-07-22

**Authors:** Xiao-juan Yu, Nan Hu, Su-xia Wang, Fu-de Zhou, Ming-hui Zhao

**Affiliations:** 1 Renal Division, Department of Medicine, Peking University First Hospital, Beijing, 100034 People’s Republic of China; 2 Institute of Nephrology, Peking University, Beijing, 100034 People’s Republic of China; 3 Renal Pathology Center, Institute of Nephrology, Peking University, Beijing, 100034 People’s Republic of China; 4 Key Laboratory of Renal Disease, Ministry of Health of China, Beijing, 100034 People’s Republic of China; 5 Key Laboratory of CKD Prevention and Treatment, Ministry of Education of China, Beijing, 100034 People’s Republic of China; 6 Laboratory of Electron Microscopy, Pathological Centre, Peking University First Hospital, Beijing, 100034 People’s Republic of China; 7 Peking-Tsinghua Center for Life Sciences, Beijing, People’s Republic of China

**Keywords:** MPGN, Monoclonal gammopathy, MGRS, PGNMID

## Abstract

**Background:**

Proliferative glomerulonephritis with monoclonal Immunoglobulin (G) deposits (PGNMID) is a rare kind of MGRS with intact monoclonal IgG deposition. Seventy percent of PGNMID patients were negative for M-spike.

**Case presentation:**

A 51-year-old Chinese woman presented with 16-month history of chronic nephritic syndrome. Her first biopsy showed a MPGN pattern, and the IF showed polyclonal IgG deposition but with IgG3λ dominance, MGRS was highly suspected. But the serum/urine IFE and bone marrow examination was negative for monoclonal gammopathy. She was treated with RAS inhibitors, and monitored carefully in the outpatient clinic. When the proteinuria was not controlled by RAS inhibitors, immunosuppressive agents were initiated. The second biopsy was done due to her acute kidney injury 9 months later, showing a MPGN pattern with acute tubulointerstitial disease, but the IF showed monoclonal IgG3λ deposition. The κ light chain, IgG1, IgG2 and IgG4 were absent. Electron microscopic examination revealed electron-dense deposits in the mesangial, subendothelial and subepithelial area which is the same as the first renal biopsy. The final diagnose of this patient was PGNMID (IgG3λ) with non-organized deposits. Repeated serum/urine IFE and free light chain still failed to identify monoclonal gammopathy. The patient was treated with steroid and cyclophosphamide, and her serum creatinine decreased.

**Conclusions:**

Some of the PGNMID patients may be derived from polyclonal immune complex mediated glomerulonephritis.

## Background

Membranoproliferative glomerulonephritis (MPGN) describes a pathologic pattern characterized by mesangial hypercellularity and matrix proliferation, as well as remodeling of capillary wall with double contours. MPGN is further classified based on immunofluorescence (IF) staining and pathogenesis [1]. Polyclonal immunogloblin and complement deposition indicates autoimmune diseases or chronic infections. Monoclonal immunoglobulin deposition indicates lymphoplasmoproferative disease. Strong positive staining of C3 with scanty or none of the immunoglobulins, C4 or C1q indicates C3 glomerulopathy (C3G). Similarly, strong positive staining of C4 with scanty or none of the immunoglobulins, C3 or C1q indicates C4 glomerulopathy [2]. None of the immunoglobulin or complement deposition indicates microangiopathy. Accurate IF staining on frozen and paraffin tissue is of vital importance for identifying the causes of MPGN and issuing the treatment.

MPGN with monoclonal immunoglobulin deposition can be seen in monoclonal gammopathy of renal significance (MGRS), multiple myeloma, and lymphoma/leukemia. MGRS is a recently defined group of diseases that the kidney injuries are either directly caused by the deposition of monoclonal immunoglobulin or indirectly via other mechanisms (e.g. autoantibodies to complement factor H) mediated glomerulonephritis (C3G), meanwhile excluding patients with malignancies (e.g. multiple myeloma) [3–5]. Proliferative glomerulonephritis with monoclonal Immunoglobulin G (IgG) deposits (PGNMID) is a rare kind of MGRS with intact monoclonal IgG (single light-chain isotype and single γ heavy chain subtype) deposition [6]. Here we report a case of MPGN with first renal biopsy showing polyclonal IgG deposition with IgG3λ dominance, and 9 months later the second renal biopsy showing monoclonal IgG3λ deposition alone and the patient was finally diagnosed as PGNMID.

## Case presentation

A 51-year-old Chinese woman presented with 16-month history of proteinuria and hypertension (160/90 mmHg) which was noticed during a routine examination. She was treated with Valsartan and blood pressure was controlled around 120/70 mmHg. Three months before admission, her urinary protein excretion was 2.12 g/d, serum albumin 36.4 g/L (normal range: 40–55 g/L), and serum creatinine 0.72 mg/dl (normal range: 0.50–1.50 mg/dl). One month before admission, her urinary protein excretion increased to 4.6 g/d, and serum creatinine increased to 1.16 mg/dl.

The patient was discovered Hepatitis C virus (HCV) infection 3 months prior to her admission, but not knowing how she got the infection. HCV-RNA was negative at that time and she did not receive any antiviral treatment. Family history was of no significance.

On admission, her blood pressure was 131/84 mmHg, temperature 36.7 °C, heart rate 75/min, and respiratory rate 18/min. There was mild edema around the eyelid, and there was no organomegaly. Other physical examinations were normal.

After admission, urine dipstick revealed proteinuria 2+. Urine sediment analysis revealed red blood cell 6 to 8 cells per high power field without white blood cell. Urinary protein excretion was 4.03 to 4.49 g/24 h. The urine albumin creatinine ratio was 2512.42 mg/gCr (normal range: < 30 mg/gCr). Her serum total protein was 58.4 g/L (normal range: 65–85 g/L), albumin was 35.3 to 29.7 g/L, and serum creatinine was 0.87 mg/dl to 1.03 mg/dl with estimated glomerular filtration rate (eGFR) of 64.33 to 63.39 ml/min/1.73m^2^. Her white blood cell (WBC) was 6.10 × 10^9^ cells/L (normal range: 3.5–9.5 × 10^9^ cells/L), hemoglobin was 101 g/L (normal range: 115–150 g/L) and platelet was 196 × 10^9^ cells/L (normal range: 125–300 × 10^9^ cells/L). Serum anti-HCV antibody was still positive and serum HCV-RNA was undetectable. Serum cryoglobulin was negative. She was negative for hepatitis B surface antigen (HBsAg), anti- human immunodeficiency virus (HIV) and Treponema pallidum antibody (TP-Ab). Other laboratory data revealed serum immunoglobulin (Ig) G was 5.42 g/L (normal range: 7.23–16.85 g/L), IgA was 1.37 g/L (normal range: 0.69–3.82 g/L), and IgM was 0.60 g/L (normal range: 0.63–2.77 g/L). Serum C3 level was 0.674 g/L (normal range: 0.60–1.50 g/L), and C4 level was 0.154 g/L (normal range: 0.12–0.36 g/L). Serum and urine immunofixation electrophoresis (IFE) did not identify monoclonal immunoglobulins. Anti-nuclear antibodies, anti-neutrophil cytoplasmic antibodies and anti- phospholipase A2 receptor (PLA2R) antibodies were all negative. Echocardiography and abdominal ultrasound were normal.

**Fig. 1:**
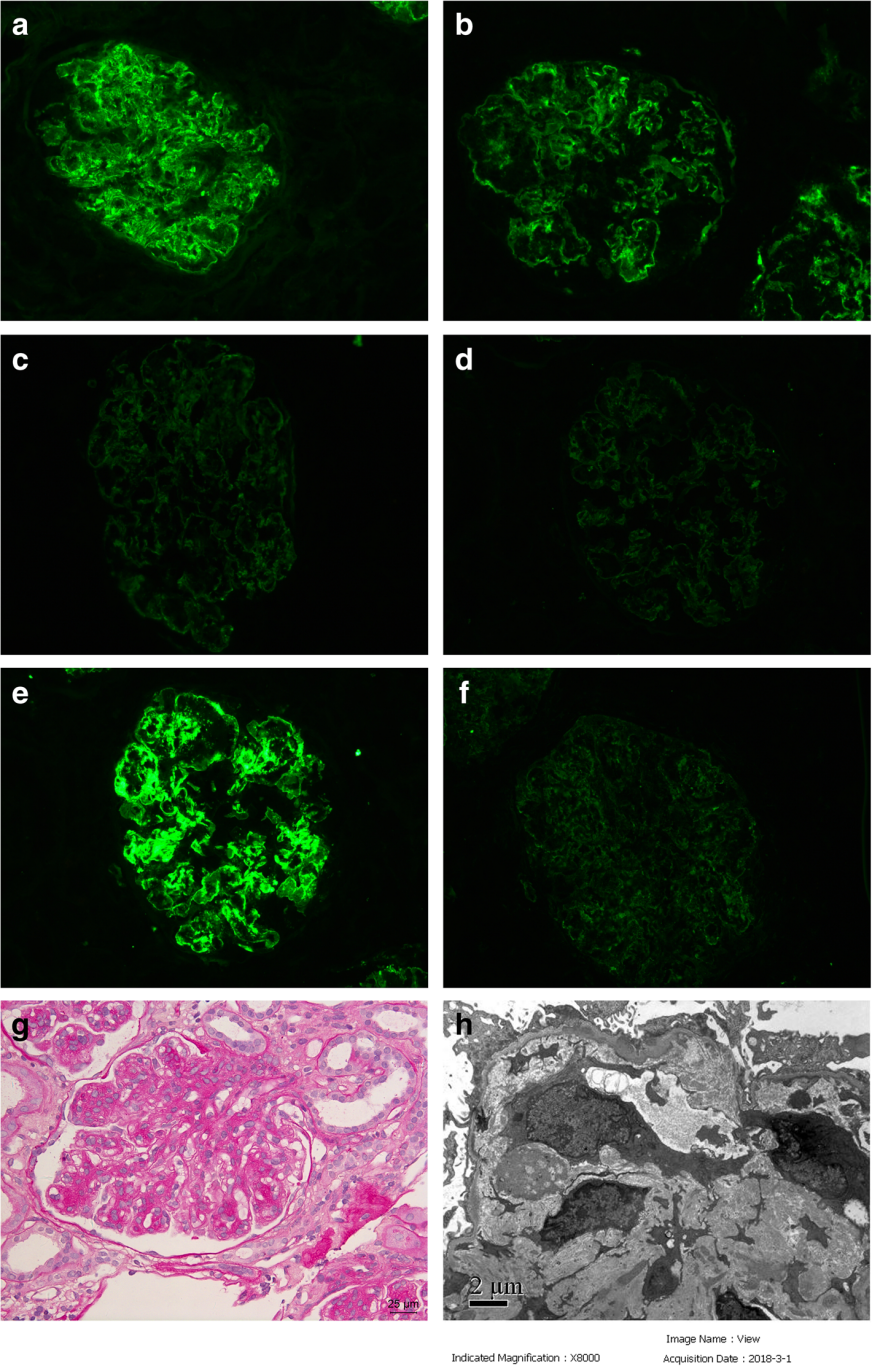
Pathlogical findings of the first renal biopsy. **a** Frozen tissue on IF showed granular IgG deposition along the capillary wall and in the mesangial area. (× 200). **b** IF showed granular C3 deposition along the capillary wall and in the mesangial area. (× 200). **c** IF showed granular C4d deposition along the capillary wall and in the mesangial area. (× 200). **d** IF showed granular κ light chain deposition along the capillary wall and in the mesangial area. (× 200). **e** IF showed granular λ light chain deposition along the capillary wall and in the mesangial area. (× 200). **f** IF showed granular IgG3 deposition along the capillary wall and in the mesangial area. (× 200). **g** Light microscopy showed a glomerular MPGN pattern. (periodic acid-Schiff staining, ×400). **h** Electron microscopy showed the electron-dense deposits in the mesangial, subendothelial and subepithelial areas of glomeruli. (× 8000)

The patient underwent the first renal biopsy on February 26th, 2018. Direct immunofluorescence (IF) examination of frozen renal tissue revealed IgG +++, IgM +, C3 +++, C1q trace, C4d +++, κ++, λ++++, IgG1 +, IgG2 ++, IgG3 +++, IgG4 +, which were deposited along the capillary wall and mesangial area of the glomeruli (Fig. 1a-f). Light microscopic examination showed that 10/47 glomeruli were globally sclerosed. Other glomeruli showed a MPGN pattern with severe mesangial cell and matrix proliferation with nodular lesions, thickening of the capillary wall and double contour formation (Fig. 1g). Congo red staining for amyloid was negative. Electron microscopic examination revealed electron-dense deposits in the mesangial, subendothelial and subepithelial area. Subendothelial edema was observed in segmental capillary loops (Fig. 1h). Hence the pathological diagnosis was immune-complex mediated MPGN due to polyclonal immunoglobulins deposition.

To explore the etiology and pathogenesis of MPGN, the differential diagnosis and further examinations were performed. Autoimmune diseases were excluded as the absence of antoantibodies and relevant clinical manifestations. The anti-HCV antibodies were positive in the serum, but the serum HCV-RNA was negative, as were the serum cryoglobulin, serum C3 and C4 and HCV antigen staining on the renal tissue, excluding the diagnosis of HCV related MPGN. Other chronic infections were not identified. According to the IF findings by dominant IgG3 and λ light chain, monoclonal gammopathy associated MPGN was suspected. Bone marrow aspiration smear revealed 1.5% plasma cells. Bone marrow biopsy showed a few plasma cells with normal change. CD38 positive cells accounted for 0.28% of bone marrow cells, but no evidence of monoclonal light chain restricted expression as determined by flow cytometry and fluorescent in situ hybridization (FISH). She was treated with olmesartan 40 mg/d, ramipril 10 mg/d, amlodipine 10 mg/d and metoprolol 25 mg/d, blood pressure was controlled around 120/70 mmHg. She did not receive any anti-HCV treatment.

Six months after renal biopsy, her proteinuria increased to 5.57 g/d, with serum albumin 31.8 g/L, serum creatinine 1.29 mg/dl. Therefore, mycophenolate mofetil and tripterygium wilfordii were prescribed. One month later, her proteinuria decreased to 2.47 g/d with a serum albumin 27.7 g/L, serum creatinine 0.97 mg/dl. However, her serum creatinine increased to 1.96 mg/dl, and a repeated renal biopsy was performed 9 months later in November 19th, 2018.

**Fig. 2:**
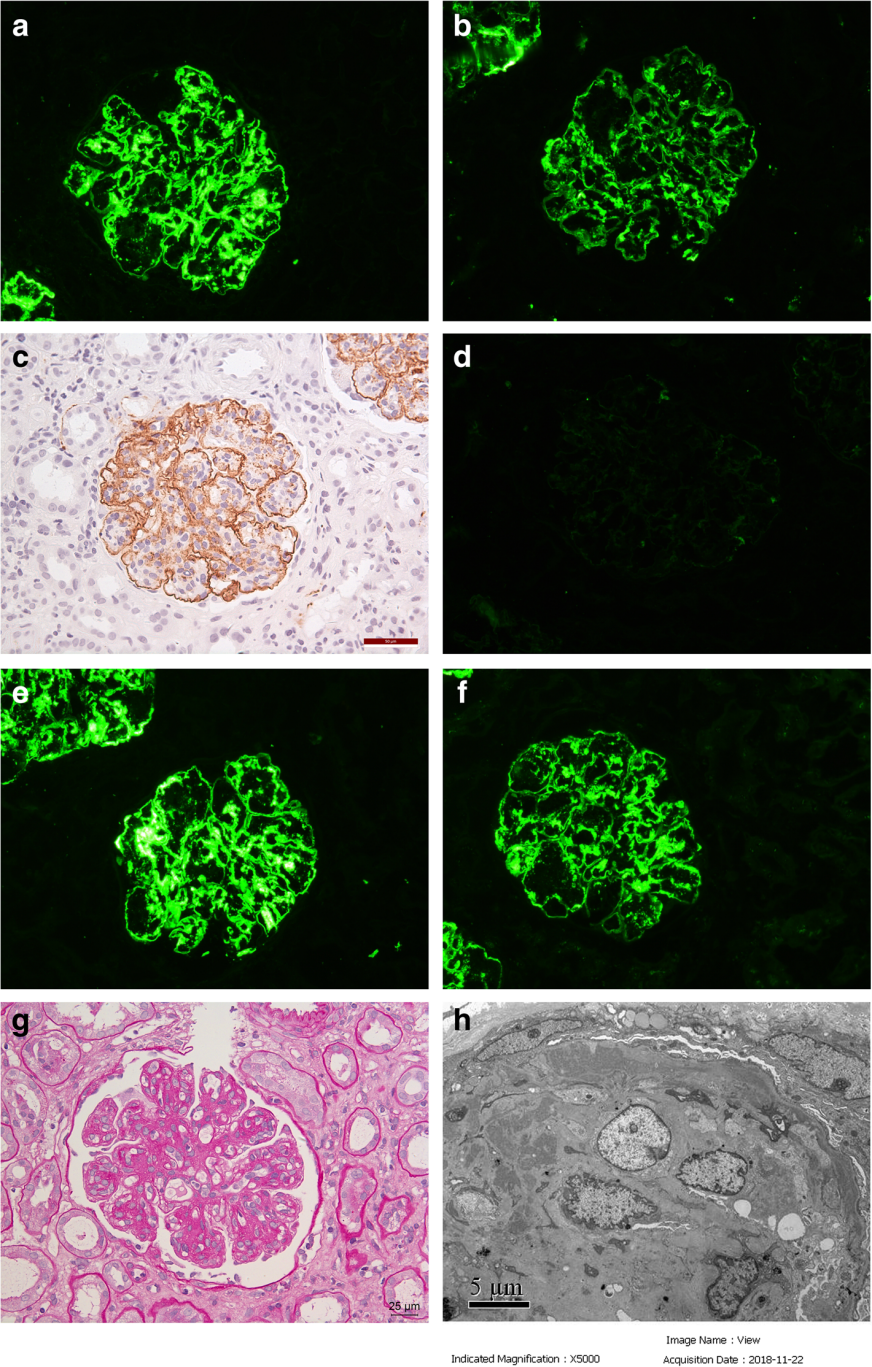
Pathological findings of the second renal biopsy. **a** Frozen tissue on IF showed granular IgG deposition along the capillary wall and in the mesangial area. (× 200). **b** IF showed granular C3 deposition along the capillary wall and in the mesangial area. (× 200). **c** Immunohistiochemistry on paraffin tissue showed granular C4d deposition along the capillary wall and in the mesangial area. (×400). **d** IF showed trace κ light chain deposition. (× 200). **e** IF showed strong granular λ light chain deposition along the capillary wall and in the mesangial area. (× 200). **f** IF showed strong granular IgG3 deposition along the capillary wall and in the mesangial area. (× 200). **g** Light microscopy showed a MPGN pattern of the glomeruli. (periodic acid-Schiff staining, ×400). **h** Electron microscopy showed the electron-dense deposits in the mesangial, subendothelial and subepithelial areas of the glomeruli. (× 5000)

IF examination of frozen renal tissue revealed IgG ++++, C3 +++, C1q negative, C4d +++ (immunohistochemistry), κ trace, λ++++, IgG1 negative, IgG2 negative, IgG3 +++,IgG4 negative, which were deposited along the capillary wall and mesangial area of the glomeruli (Fig. 2a-f). Light microscopic examination showed that 2/20 glomeruli were globally sclerosed. Other glomeruli showed a MPGN pattern(Fig. 1g). More prominent tubular atrophy and interstitial fibrosis than the first time, with focal loss of brush border of the tubular epithelial cells. There was moderate interstitial infiltration of lymphocytes, monocytes and a few eosinophils. Electron microscopic examination revealed electron-dense deposits in the mesangial, subendothelial and subepithelial area. (Fig. 2h). The patient was diagnosed with proliferative glomerulonephritis with monoclonal IgG3λ deposition (PGNMID). Further testes showed serum free κ chain was 16.8 mg/L (normal range: 3.30–19.40 mg/L), free λ chain was 25.3 mg/L (normal range: 5.71–26.3 mg/L), and the κ/λ ratio was 0.664 (normal range: 0.26–1.65). Serum and urine IFE were still negative for monoclonal immunoglobulin. Repeated serum anti-HCV antibody detection was negative without anti-HCV treatment.

Therapy and follw-up: The patient was treated with prednisone (40 mg/d) and cyclophosphamide (100 mg/d), her serum creatinine decreased to 1.47 mg/dl and remained stable until March 2019. Then she suffer an episode of herpes zoster, cyclophosphamide was discontinued, and the patient’s serum creatinine increased to 2.54 mg/dl.

## Discussion and conclusions

The patient we reported in the current study showed a MPGN pattern in two consecutive renal biopsies. Based on the IF of the first biopsy, this patient was first diagnosed with polyclonal immunoglobulin mediated MPGN, but the IF showed IgG3λ dominant deposition, MGRS was highly suspected. But the serum/urine IFE and bone marrow examination was negative for monoclonal gammopathy. She was treated with RAS inhibitors, and monitored carefully in the outpatient clinic. When the proteinuria was not controlled by RAS inhibitors, the patient was treated with immunosuppressive agents. The second biopsy was done due to her acute kidney injury. The IF of the second biopsy showed monoclonal IgG3λ deposition which was different from the first biopsy. Κ light chain, IgG1, IgG2 and IgG4 became absent. The final diagnose of this patient was PGNMID (IgG3λ) with non-organized deposits. Repeat serum/urine IFE and free light chain still failed to identify monoclonal gammopathy. These two renal biopsies were both done in our hospital and the IF was processed by the same operator with a similar technology and analytical approach.

PGNMID is a rare form of MGRS characterized by intact monoclonal IgG deposition, the most common type was IgG3κ (47%~ 53.1%), followed by IgG1κ (21.9%~ 26%), IgG3λ (5%~ 12.5%), IgG1λ (6.3%~ 16%), and IgG4κ(0%~ 5%) [7, 8]. MPGN (57%) and endocapillary proliferative glomerulonephritis (35%) were the most common light microscopy pathologic patterns [7]. However, Guiard E et al. reported that both of membranous nephropathy and MPGN were the two common pathologic patterns [8]. Other studies reported cases of MN correlated with IgG1 [7–9], and MPGN correlated with IgG3. On EM, the electron dense deposits are very similar to polyclonal immune-complex mediated glomerulonephritis which can be deposited in the subendothelial, mesangial, occasionally subepithelial and/or intramembranous areas. These deposits mostly showed non-organized granular pattern, but organized structures, including fibrils (15-21 nm in diameter) and lattice-like structures (15 nm) were also reported in 32.4% of PGNMID patients, but very focally [7, 8]. Seventy percent of the PGNMID patients failed to identify monoclonal immuoglobulins or MIg-producing cells in the serum, urine and bone marrow [7, 8]. Rare patients (3%) developed monoclonal spike during follow up [7]. Malignant diseases (e.g. multiple myeloma, lymphoma, leukemia) are uncommon (3%~ 26%) in PGNMID [7, 8].

The pathogenesis of PGNMID remains elusive. It was speculated that monoclonal IgG was produced during immune response to extrinsic or intrinsic antigens. These monoclonal IgG had the ability of rapid self-aggregation which was favored by its intrinsic physical properties, high avidity for glomeruli and could rapid deposition in glomeruli via passive entrapment and/or interaction with negatively charged glomerular constituents. This monoclonal IgG was so little in serum/urine that it can not be detected by SPEP/UPEP/IFE [7]. There are four IgG subclasses. IgG3 is the most nephritogenic subclass due to the following reasons: (1) most positively charged; (2) highest molecular weight; (3) self-aggregation in the glomerular capillary via a specific Fc-Fc interaction; (4) the highest capability of activating complements, mediating inflammation which may explain the high frequency of C3 (97.3%), C4d and C1q (63.4%). These properties explain the highest frequency of IgG3, and lack of IgG4. Based on this case report, it is suspected that some of the PGNMID patients may be derived from polyclonal MPGN. The IgG1, IgG2, IgG4 and maybe IgG3κ were digested or cleaned from the glomeruli leaving monoclonal IgG3λ behind due to the special properties of IgG3. This may explain the lack of monoclonal gammopathy in the serum, urine or bone marrow in most of the PGNMID-IgG3 patients (19/21, 90%), while most of the M-spike positive patients (7/11, 64%) were IgG1 and IgG2 positive [7]. The similarity of the electron dense deposits with polyclonal immune complex glomerulonephritis, and IgG3λ-PGNMID was reported in 9 and 17 years old patients, respectively [10, 11]. But in some cases when there is monoclonal IgG in the serum and/or urine (especially IgG1 and IgG2), the patients maybe truly be diagnosed as MGRS.

HCV can cause renal disease via mixed cryogloblulinemia, MPGN, membranous nephropathy and polyarteritis nodosa. For our patient, HCV associated MPGN in the absence of cryoglobulinemia should be suspected. HCV-MPGN first manifests as polyclonal immunoglobulins deposition, but it can also stimulate monoclonal B cell proliferation leading to monoclonal-MPGN. However, at the time of the first biopsy, the patient’s serum HCV-RNA was negative, as were the tissue staining for HCV-antigen. At the second biopsy, her serum anti-HCV antibodies were negative, no sign of monoclonal spike in the serum or urine and lymphoproliferative diseases, which altogether did not support HCV associated MPGN. However, we could not explain how the patient’s anti-HCV antibody turning negative without anti-viral treatment and whether this phenomenon is associated with MPGN lesion.

The prognosis of PGNMID is poor, during a mean follow up of 30.3 months, 21.9% patients progressed to ESRD, and 37.5% had persistent renal dysfunction. Only 3.7% M-spike negative patient at presentation developed M-spike during follow up, and none of patients with M-spike at presentation developed multiple myeloma or lymphoma. Higher serum creatinine level, higher percentage of global glomerulosclerosis, greater degree of tubular atrophy, interstitial fibrosis and arteriosclerosis were associated with ESRD, which is very similar to other kidney diseases. PGNMID may recur early after renal transplantation (5–19 months) [12]. The treatment of PGNMID is controversial due to limited cases and uncertain pathogenesis. For M-spike negative patients, non-nephrotic syndrome patients are suggested with conservative treatment including RAS inhibitors. Steroids and cyclophosphamide are suggested in nephrotic syndrome, RAS inhibitor treatment failing patients, a decreased GFR or biopsy features suggestive of progression (e.g. crescents). Rituximab alone may have better remission rate and better tolerance than steroids and cyclophosphamide [8, 13]. Chemotherapy including bortezomib may be reasonable for M-spike positive patients. Our patient was first treated with RAS inhibitors, and MMF was added when she progressed to nephrotic syndrome. After the second biopsy, the patient was given steroids and cyclophosphamide to treat both the PGNMID and interstitial nephritis. The serum creatinine decreased, but proteinuria sustained, probably due to the limited follow-up time. The patient has been followed up closely in the out-patient clinic, including monitor the M-spike.

We report a MPGN patient with 2 consecutive renal biopsies within 9 months. IF of the first biopsy showed polyclonal immunogloblin deposition but IgG3λ dominance. IF of the second biopsy showed monoclonal IgG3λ deposition. The final diagnose was PGNMID (IgG3λ) with non-organized deposits. The patient was negative for monoclonal gammopathy during the entire follow up time. It is suspected that some of the PGNMID patients (especially IgG3) may be evolved from polyclonal immune complex mediated glomerulonephritis.

## Data Availability

All data generated or analyzed during this study are included in this published article.

## Abbreviations

ACR: albumin creatinine ratio
C1q: complement 1q
C3: complement 3
C3G: C3 glomerulopathy
C4: complement 4
CD: cluster of differentiation
eGFR: estimated glomerular filtration rate
ESRD: end stage renal disease
FISH: fluorescent in situ hybridization
HBsAg: hepatitis B surface antigen
HCV: hepatitis C virus
HIV: human immunodeficiency virus
IF: immunofluorescence
IFE: immunofixation electropheresis
IgA: immunoglobulin A
IgG: immunoglobulin G
IgM: immunoglobulin M
MGRS: monoclonal gammopathy of renal significance
MIg: monoclonal immunoglobulin
MMF: mycophenolate motetil
MPGN: membranoproliferative glomerulonephritis
M-spike: monoclonal-spike
PGNMID: Proliferative glomerulonephritis with monoclonal Immunoglobulin (G) deposits
PLA2R: phospholipase A2 receptor
RAS: renin-angiotensin system
RNA: ribonucleic acid
SCr: serum creatinine
SPEP: serum protein electrophoresis
TP-Ab: Treponema pallidum antibody
UPEP: urine protein electrophoresis

## References

[1] Sethi S, Fervenza FC (2012). Membranoproliferative glomerulonephritis--a new look at an old entity. N Engl J Med.

[2] Sethi S, Sullivan A, Smith RJ (2014). C4 dense-deposit disease. N Engl J Med.

[3] Bridoux F (2015). Diagnosis of monoclonal gammopathy of renal significance. Kidney Int.

[4] Leung N, et al. Publisher correction: the evaluation of monoclonal gammopathy of renal significance: a consensus report of the international kidney and monoclonal Gammopathy research group. Nat Rev Nephrol. 2018. PMID:30568288. 10.1038/s41581-018-0102-7 PMC7608309 30568288

[5] Leung N (2019). The evaluation of monoclonal gammopathy of renal significance: a consensus report of the international kidney and monoclonal Gammopathy research group. Nat Rev Nephrol.

[6] Nasr SH (2004). Proliferative glomerulonephritis with monoclonal IgG deposits: a distinct entity mimicking immune-complex glomerulonephritis. Kidney Int.

[7] Nasr SH (2009). Proliferative glomerulonephritis with monoclonal IgG deposits. J Am Soc Nephrol.

[8] Guiard E (2011). Patterns of noncryoglobulinemic glomerulonephritis with monoclonal Ig deposits: correlation with IgG subclass and response to rituximab. Clin J Am Soc Nephrol.

[9] Bridoux F (2002). Fibrillary glomerulonephritis and immunotactoid (microtubular) glomerulopathy are associated with distinct immunologic features. Kidney Int.

[10] Tamura T (2018). A case of recurrent proliferative glomerulonephritis with monoclonal IgG deposits or de novo C3 glomerulonephritis after kidney transplantation. Nephrology (Carlton).

[11] Torrealba J, Gattineni J, Hendricks AR (2018). Proliferative glomerulonephritis with monoclonal immunoglobulin G lambda deposits: report of the first pediatric case. Case Rep Nephrol Dial.

[12] Wen J (2018). Clinicopathological analysis of proliferative glomerulonephritis with monoclonal IgG deposits in 5 renal allografts. BMC Nephrol.

[13] Maan Dipesh, Clark Barbara, Bunker Mark, Arora Swati (2018). Successful management of proliferative glomerulonephritis with monoclonal immune deposits with combined immunosuppressive therapy. BMJ Case Reports.

